# Conservative management of a grade V injury to an ectopic pelvic kidney following blunt trauma to the lower abdomen: a case report

**DOI:** 10.1186/1752-1947-4-224

**Published:** 2010-07-24

**Authors:** Aaron B Becker, Mirza B Baig, Adam M Becker

**Affiliations:** 1 University of Toledo Medical Center, Department of Urology, Dowling Hall 2nd floor, 3065 Arlington Avenue, Toledo, OH 43614, USA

## Abstract

**Introduction:**

Ectopic pelvic kidneys represent an anatomic variant that remains clinically asymptomatic in most patients. While there is some literature to suggest that ectopic kidneys may be more predisposed to blunt trauma injuries, there are few examples to guide the management of these injuries. To our knowledge, we present the first case of a grade V renal injury to an ectopic pelvic kidney managed successfully with conservative measures.

**Case Presentation:**

We present a case of grade V renal injury to an ectopic pelvic kidney in a 21 year-old African-American male. The clinical and radiographic findings are presented, along with the patient's conservative hospital course.

**Conclusion:**

We suggest that management of grade V renal injuries to ectopic pelvic kidneys can be treated similarly to that of kidneys in normal anatomic position. Conservative measures may be considered in properly selected patients.

## Introduction

Ectopic pelvic kidneys occur with a reported incidence of between 1 in 500 to 1 in 1200. Although pelvic kidneys are associated with anomalies including hydronephrosis and vesicoureteral reflux, most are clinically asymptomatic [1]. Pelvic kidneys are, however, more prone to blunt trauma injury [2]. Little literature exists regarding the optimal management of blunt trauma injury in ectopic pelvic kidneys. We report a case of a grade V renal injury to an ectopic pelvic kidney managed successfully with conservative measures.

## Case Presentation

**Figure 1:**
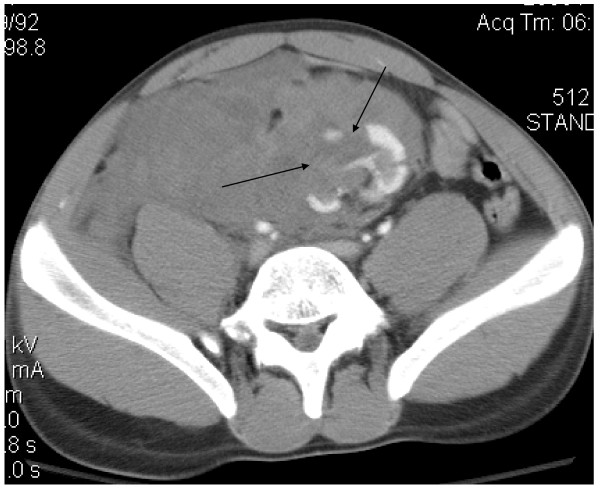
**Computerized tomography of the abdomen and pelvis with intravenous contrast demonstrating an ectopic pelvic kidney with multiple deep lacerations (indicated by arrows), and a large retroperitoneal hematoma displacing the kidney to the left lower abdomen**.

**Figure 2:**
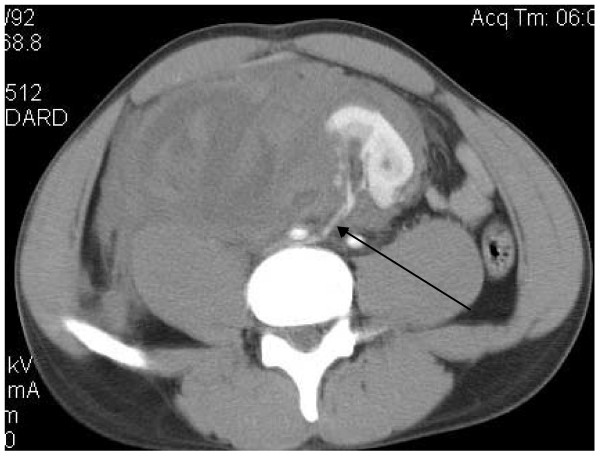
**Computerized tomography of the abdomen and pelvis with intravenous contrast showing an intact right renal artery originating from the right common iliac artery (indicated by an arrow)**.

A 21 year-old African-American male presented to the emergency room with complaints of right-sided abdominal pain and gross hematuria following blunt trauma to the abdomen. The patient had been kneed in the right lower quadrant. The physical examination revealed a hemodynamically stable male in acute distress, with marked tenderness in the right lower quadrant. A foley catheter was placed with return of gross hematuria. Laboratory examination revealed a hemoglobin of 12 g/dL as well as a serum creatinine of 1.4 mg/dL. Computerized tomography (CT) of the abdomen and pelvis with intravenous contrast demonstrated a normally positioned left kidney, multiple deep lacerations to an ectopic pelvic kidney suggestive of a grade V injury, and a large retroperitoneal hematoma (Figure 1). Both kidneys appeared equal in size, measuring approximately 11 cm in length and 5 cm in width. The main arterial supply to the ectopic kidney appeared intact, with a right renal artery originating from the right common iliac artery (Figure 2).

In light of the patient's hemodynamic stability, the patient was admitted to the intensive care unit and treated conservatively with aggressive fluid resuscitation, serial hemoglobin levels, and bed rest. The patient experienced a prolonged hospital course secondary to hematuria, hospitalized for a total of nineteen days. The patient remained hemodynamically stable throughout his hospitalization with a serum creatinine within normal limits, but required six total units of packed red blood cells for anemia with hemoglobins near 8 g/dL. The patient's hematuria resolved on hospital day sixteen, and the patient was then ambulated with no further hematuria or anemia noted.

**Figure 3:**
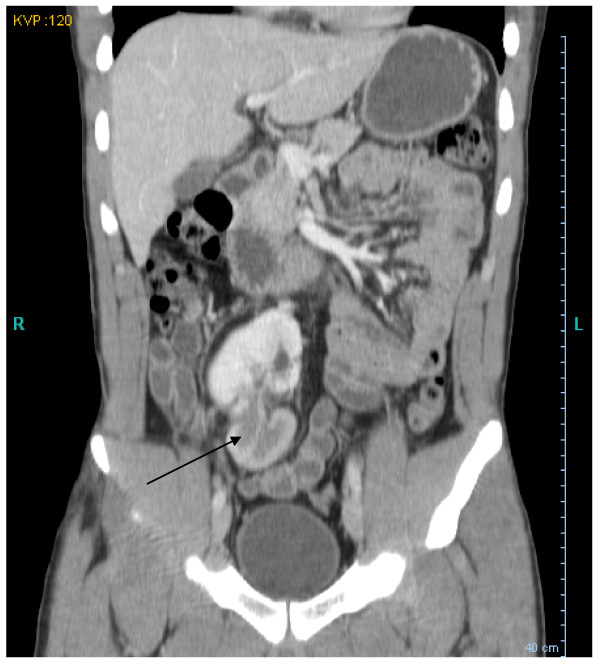
**Computerized tomography of the abdomen and pelvis with intravenous contrast demonstrating resolution of the retroperitoneal hematoma, return of the pelvic kidney to its anatomical position in the right pelvis, as well as persistent perfusion defects in the lower pole (indicated by an arrow)**.

Three months following the trauma, imaging revealed resolution of the retroperitoneal hematoma, return of the pelvic kidney to its anatomical position in the right pelvis, and perfusion defects in the lower pole, likely representing persistent renal injury (Figure 3). At follow-up, serum creatinine was 1.3 mg/dL.

## Discussion

Kidney injuries occur in approximately 10% of blunt abdominal trauma cases. Abnormal kidneys, including ectopic kidneys, may be more predisposed to injury as they are often located in a less-protected anatomical position in the retroperitoneum. In a meta-analysis by Schmidlin *et al*., ectopic kidneys constituted 7% of cases involving abnormal kidneys. Abnormal kidneys in total represented 7% of all blunt renal trauma cases, and included such conditions as renal cysts, hydronephrosis, and renal tumors. Furthermore, it was found that trauma to abnormal kidneys, including ectopic kidneys, is more frequently caused by low velocity impacts and has a lower rate of associated trauma to other abdominal organs [2].

High-grade renal injuries including grade IV and V injuries comprise the minority of renal trauma cases, with grade V injuries representing only 5% of blunt renal trauma cases. Grade IV injuries can be defined as deep parenchyma lacerations involving the collecting system, as well as vascular injuries to a segmental renal arterial branch. Grade V injuries can be defined as multiple deep lacerations into the renal parenchyma resulting in a shattered kidney, as well as devascularizing injuries to the renal pedicle, or avulsion of the main renal artery [3]. In this case the patient presented with multiple deep lacerations to the parenchyma of the ectopic pelvic kidney consistent with a grade V injury.

While classically grade V renal injuries have been managed surgically, the conservative management of grade IV injuries in the setting of blunt trauma has been well established. In a meta-analysis of 16 published reports, 90% of 324 grade IV blunt renal injuries could be managed conservatively, with 12.6% requiring delayed surgical intervention and 4.6% requiring nephrectomy [4]. Despite the traditional standard of operative intervention in grade V injuries, current literature suggests that many of these injuries may be managed conservatively, particularly in those who remain hemodynamically stable upon presentation. Altman *et al*. reported a series of 13 patients with grade V renal injuries, of whom six were treated conservatively with fewer intensive care unit days (4.3 versus 9.0), significantly fewer transfusion units (2.7 versus 25.2), and fewer complications versus those undergoing operative management [5]. Proponents of conservative measures in an effort to avoid a trauma nephrectomy note a lower creatinine clearance in the peri-injury period among those undergoing trauma nephrectomies versus those with no renal injury, as well as an increase in mortality (8% to 16%) and acute renal failure (7% versus 11%) [6]. However, some attribute these differences to associated injuries and age, rather then renal removal itself.

Despite efforts to manage select high-grade blunt renal injuries nonoperatively, there are many clinical scenarios representing absolute indications for operative intervention. These include shock secondary to renal bleeding, expanding retroperitoneal hematoma, transfusion requirements exceeding 3 U/day of packed red blood cells associated with hemodynamic instability, renal pelvic or ureteral injury, and certain renovascular conditions such as renal artery stenosis. In addition, active extravasation of contrast-enhanced blood on CT may represent a subset of patients who may warrant operative intervention, as it likely represents brisk bleeding in a patient who may not yet be hemodynamically-stable [7]. Jeffrey *et al*. found that among 18 patients with active extravasation, 50% required open surgery, 28% angiography, and 22% bled to death or required multiple blood transfusions [8]. Criticisms of conservative management for high-grade renal injuries have also focused on the delayed complications which may theoretically be avoided with nephrectomy or renorrhaphy. Chief among these is urinary extravasation, although most (74% to 87%) will resolve spontaneously with conservative measures. Additional potential complications include hypertension, whose incidence varies widely from 0.25% to 55%, as well as arteriovenous fistula and pseudoaneurysm, both considered rare complications. Finally delayed bleeding must be considered, present in up to 20% of cases[7].

Although ectopic kidneys are more susceptible to blunt trauma injuries, there is little current literature on the management of Grade V injuries to an ectopic kidney. Schmidlin *et al*. report two cases of blunt renal trauma to a ectopic kidneys, of which one required operative intervention. This study did not, however, describe the extent of the injuries, nor the indication for surgical intervention in that individual case [2]. Our patient represents an interesting case of an isolated Grade V renal injury who responded favorably to conservative measures.

## Conclusion

We present an interesting case of a grade V injury to an ectopic pelvic kidney, and suggest that management of these high-grade injuries to ectopic kidneys can be treated similarly to that of kidneys in a normal anatomic position. Specifically, these injuries can be managed successfully with nonoperative intervention in properly selected patients.

## Consent

Written informed consent was obtained from the patient for publication of this case report and any accompanying images. A copy of the written consent is available for review by the Editor-in-Chief of this journal.

## Competing interests

The authors declare that they have no competing interests.

## Authors' contributions

ABB participated in the design of the study and the drafting of the manuscript. MBB conceived of the study and participated in its coordination. AMB participated in the drafting of the manuscript. All authors read and approved the final manuscript.

## References

[B1] Alan J Wein, Louis R Kavoussi, Andrew Novick Campbell-Walsh urology Review Manual 2007 9 Alan Partin and Craig Peters, Philadelphia: Saunders Elsevier

[B2] Schmidlin FR Iselin CE Naimi A Rohner S Borst F Farshad M Niederer P Graber P The higher injury risk of abnormal kidneys in blunt renal trauma Scand J Urol Nephrol 1998 32 388 392 10.1080/003655998750015151 9925001

[B3] Harris AC Zwirewich CV Lyburn ID Torreggiani WC Marchinkow LO CT findings in blunt renal trauma Radiographics 2001 21 S201 S214 11598258 10.1148/radiographics.21.suppl_1.g01oc07s201

[B4] Santucci RA Fisher MB The literature increasingly supports expectant (conservative) management of renal trauma-a systematic review J Trauma 2005 59 493 503 10.1097/01.ta.0000179956.55078.c0 16294101

[B5] Altman AL Haas C Dinchman KH Spirnak JP Selective nonoperative management of blunt grade 5 renal injury J Uro 2000 164 27 31 10.1016/S0022-5347(05)67441-1 10840417

[B6] McGonigal MD Lucas CE Ledgerwood AM The effects of treatment of renal trauma on renal function J Trauma 1987 27 471 476 10.1097/00005373-198705000-00002 3573100

[B7] Broghammer JA Fisher MB Santucci RA Conservative management of renal trauma: a review Urology 2007 70 623 629 10.1016/j.urology.2007.06.1085 17991526

[B8] Jeffrey RB Jr Cardoza JD Olcott EW Detection of active intrabdominal arterial hemorrhage: value of dynamic contrast-enhanced CT AJR Am J Roentgenol 1991 156 725 729 2003435 10.2214/ajr.156.4.2003435

